# Supplementary material for: Asymmetric Synthesis of Quaternary Hydantoins via a Palladium-Catalyzed Aza-Heck Cyclization
Source: J Am Chem Soc. 2025 Nov 14;147(49):44692–8. doi: 10.1021/jacs.5c16022 (PMC12703750; doi:10.1021/jacs.5c16022)
Supplement: Supplementary file 2 [file ja5c16022_si_002.zip › All NMR FID Files/17/17_CarbonNMR/3/pdata/1/email_EM07-005-1-13C_3_1.pdf]

C13APT1024 CDCl3 /opt/nmrdata/ emeck 22

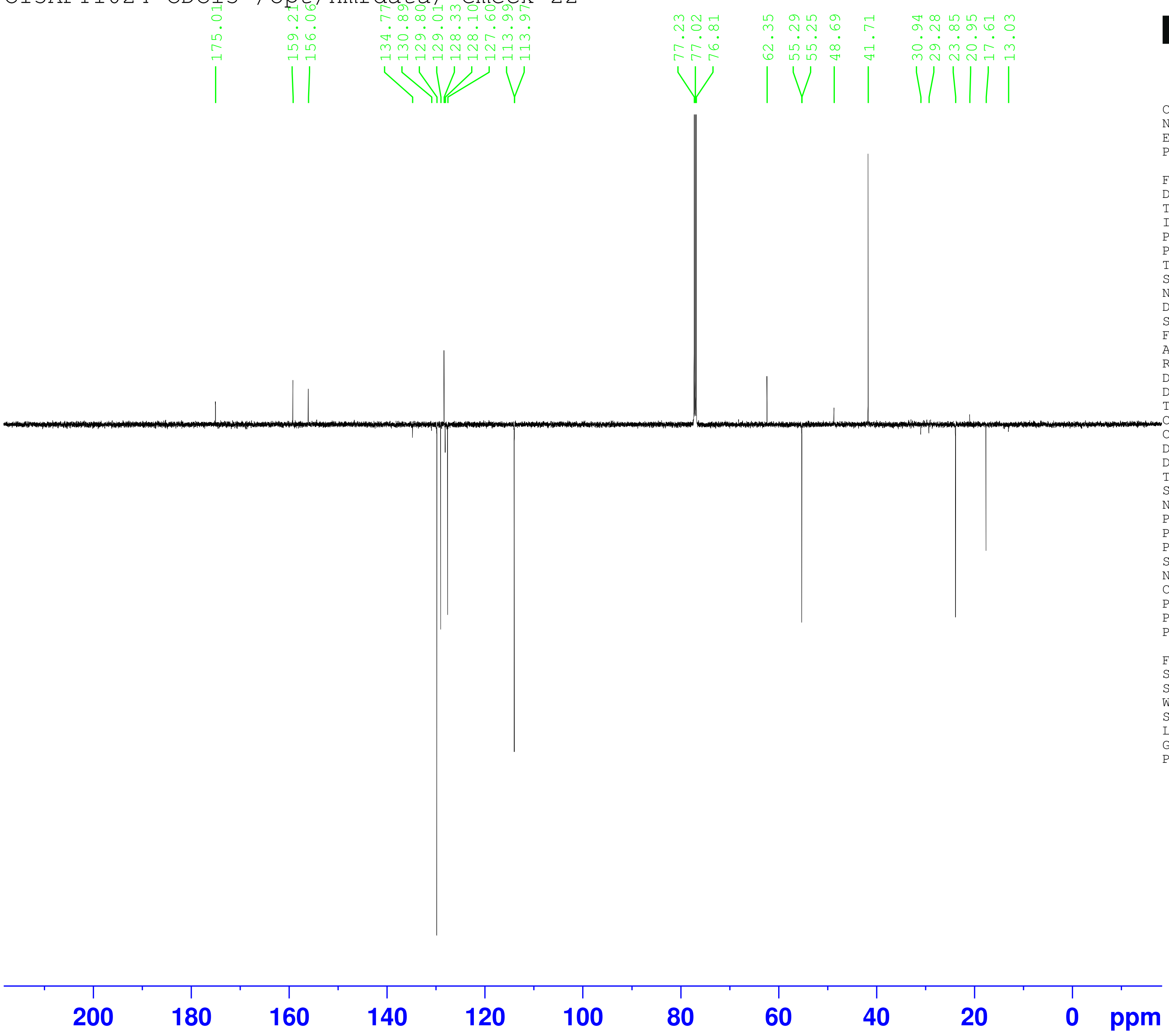


Current Data Parameters

| | |
|---|---|
| NAME | EM07-005-1-13C |
| EXPNO | 3 |
| PROCNO | 1 |

F2 - Acquisition Parameters

| | |
|---|---|
| Date_ | 20250909 |
| Time | 18.54 h |
| INSTRUM | Avance Neo600 U of DE |
| PROBHD | Z168773_0053 |
| PULPROG | jmod |
| TD | 65536 |
| SOLVENT | CDCl3 |
| NS | 1024 |
| DS | 4 |
| SWH | 35714.286 Hz |
| FIDRES | 1.089913 Hz |
| AQ | 0.9175040 sec |
| RG | 101 |
| DW | 14.000 usec |
| DE | 18.00 usec |
| TE | 298.0 K |
| CNST2 | 145.0000000 |
| CNST11 | 1.0000000 |
| D1 | 2.00000000 sec |
| D20 | 0.00689655 sec |
| TD0 | 1 |
| SFO1 | 150.9656791 MHz |
| NUC1 | 13C |
| P1 | 10.00 usec |
| P2 | 20.00 usec |
| PLW1 | 80.53800000 W |
| SFO2 | 600.3224013 MHz |
| NUC2 | 1H |
| CPDPRG2 | waltz65 |
| PCPD2 | 80.00 usec |
| PLW2 | 21.61000000 W |
| PLW12 | 0.42583000 W |

F2 - Processing parameters

| | |
|---|---|
| SI | 32768 |
| SF | 150.9505841 MHz |
| WDW | EM |
| SSB | 0 |
| LB | 1.00 Hz |
| GB | 0 |
| PC | 1.40 |
